# Anterior tibial curved cortex is a reliable landmark for tibial rotational alignment in total knee arthroplasty

**DOI:** 10.1186/s12891-017-1609-y

**Published:** 2017-06-12

**Authors:** Joong Il Kim, Jak Jang, Ki Woong Lee, Hyuk Soo Han, Sahnghoon Lee, Myung Chul Lee

**Affiliations:** 1 Department of Orthopaedic Surgery, Hallym University Kangnam Sacred Heart Hospital, 1, Singil-ro, Yeongdeungpo-gu, Seoul, 150-950 Korea; 2 Department of Orthopaedic Surgery, Seoul National University Hospital, 101 Daehak-ro, Jongno-gu, Seoul, 110-744 Korea

**Keywords:** Total knee arthroplasty, Rotational alignment, Tibial component rotation, Anatomical landmark, Anterior tibial curved cortex

## Abstract

**Background:**

Rotational alignment of the tibial component is important for long-term success of total knee arthroplasty (TKA). This study aimed to compare five axes in normal and osteoarthritic (OA) knees to determine a reliable landmark for tibial rotational alignment in TKA.

**Methods:**

One hundred twenty patients with OA knees and 40 with normal knees were included. The angle between a line perpendicular to the surgical transepicondylar axis and each of five axes were measured on preoperative computed tomography. The five axes were as follows: a line from the center of the posterior cruciate ligament (PCL) to the medial border of the patellar tendon (PCL-PT), medial border of the tibial tuberosity (PCL-TT1), medial one-third of the tibial tuberosity (PCL-TT2), and apex of the tibial tuberosity (PCL-TT3), as well as the anteroposterior axis of the tibial prosthesis along the anterior tibial curved cortex (ATCC).

**Results:**

For all five axes tested, the mean angles were smaller in OA knees than in normal knees. In normal knees, the angle of the ATCC axis had the smallest mean value and narrowest range (1.6° ± 2.8°; range, −1.7°–7.7°). In OA knees, the mean angle of the ATCC axis (0.8° ± 2.7°; range, −7.9°–9.2°) was larger than that of the PCL-TT1 axis (0.3° ± 5.5°; range, −19.7°–10.6°) (*P* = 0.461), while the angle of the ATCC axis had the smallest SD and narrowest range.

**Conclusion:**

The ATCC was found to be the most reliable and useful anatomical landmark for tibial rotational alignment in TKA.

## Background

Total knee arthroplasty (TKA) is considered a definitive treatment option for severe osteoarthritic (OA) knee [1]. Although most patients who receive TKA show successful functional outcomes, some patients have persistent knee pain, limited range of motion, and instability after TKA, eventually requiring revision TKA [2–4]. While multiple factors are relevant for achieving successful functional outcomes after TKA, the rotational alignment of the tibial component is particularly important, since malrotation can cause patellar maltracking [5–7], tibiofemoral joint instability in flexion [8–10], and premature wear of the polyethylene components, which eventually affects implant longevity [11–13].

In contrast to the transepicondylar axis (TEA), which is generally accepted as a reliable landmark for determining the rotational alignment of the femur [14–16], no gold standard has been established for determining the rotational alignment of the tibia, despite its critical relevance in the outcomes of TKA. Therefore, many anatomical landmarks on the proximal tibia have been used to determine tibial rotational alignment in TKA, including the medial one-third of the tibial tuberosity, medial border of the tibial tuberosity, apex of the tibial tuberosity, midsulcus line, and medial border of the patellar tendon [17–27]. However, these landmarks are difficult to identify after cutting the tibia and vary greatly among patients [20, 21, 28]. Indeed, Siston et al. [22] reported substantial deviations in tibial rotational alignment after TKA, which ranged from 44° of internal rotation to 46° of external rotation, depending on the surgeon’s ability.

Recently, Baldini et al. [17] proposed that the anterior tibial curved cortex (ATCC) represents a reproducible and reliable landmark for tibial rotational alignment. The approach using the ATCC as a landmark proceeds as follows: after cutting the proximal tibia, the anterior surface of the tibial baseplate is matched with the ATCC of the proximal tibia. However, the study by Baldini et al. was based on normal knees, and their measurements were not evaluated at the standard resection level for primary TKA. In addition, there has been no comprehensive comparison between the usefulness of the ATCC landmark and that of other potential landmarks. Therefore, in this study, we aimed to determine the most useful landmark for assessing tibial rotational alignment in TKA. For this reason, we considered five axes on the proximal tibia in normal and OA knees, at the standard resection level for primary TKA using preoperative computed tomography (CT). We hypothesized that, compared with the other four axes assessed, the ATCC is the most reliable landmark for optimal tibial rotational alignment, in both normal and OA knees.

## Methods

Between June and September 2010, 120 patients with OA knees and 40 with normal knees were recruited for this study. Patients who were candidates for TKA and had OA knees (grade 3 or 4 on the Kellgren-Lawrence scale) were included. Patients with inflammatory arthritis, previous open knee surgery, or infective arthritis (active or chronic) were excluded. The normal knee group comprised 40 patients without OA knees and with no ligament instability, who had been scheduled for simple meniscectomy. All patients were informed of the risk of exposure to radiation during computed tomography (CT), and written informed consent was obtained. This study was approved by our institutional review board (H-0906-044-283). Table 1 summarizes the demographic characteristics of the study participants.

**Table 1:** The demographic characteristics of the study population

	Normal knee	OA knee
Number of knees	40	120
Gender (M/F)	4:36	7:113
Mean age (year)^a^	32.1 ± 9.7	70.0 ± 7.2
BMI (kg/m^2^)^a^	25.9 ± 3.4	26.7 ± 3.2
Height (mm)^a^	163 ± 7.4	161 ± 5.4
Mechanical Tibiofemoral angle (°)^a^	varus 0.8 ± 0.4	varus 6.4 ± 3.8

^a^The values are presented as mean and standard deviation

**Fig. 1:**
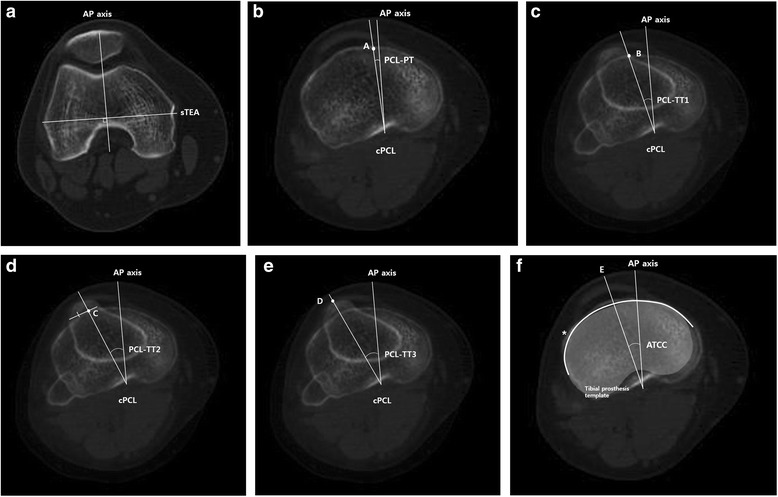
The method of angular measurement was shown. **a** The anteroposterior axis of the distal femur (AP axis), which projects perpendicular to the surgical transepicondylar axis (sTEA) that connects the most prominent points of the lateral epicondyle and sulcus of the medial epicondyle, was used as the reference axis. **b** The center of the posterior cruciate ligament was defined as cPCL. The angle PCL-PT was made by the AP axis and a line from cPCL to the medial border of the patellar tendon (**a**). **c** The angle PCL-TT1 was made by the AP axis and a line from cPCL to the medial border of the tibial tuberosity (**b**). **d** The angle PCL-TT2 was made by the AP axis and a line from cPCL to the medial one-third of the tibial tuberosity (**c**). **e** The angle PCL-TT3 was made by the AP axis and a line from cPCL to the apex of the tibial tuberosity (**d**). **f** The angle ATCC was made by the AP axis and the anteroposterior axis of the tibial prosthesis (**e**) (using tibial prosthesis template) along the anterior tibial curved cortex (asterisk)

To determine tibial rotational alignment, transverse CT scans (Siemens Somatom; Siemens Medical Solutions, Malvern, PA, USA) were obtained at 1.0- or 1.3-mm intervals from the hip to the ankle, with the knee in full extension, as described previously [24, 29]. Digital Imaging and Communications in Medicine images were processed using a three-dimensional image-reconstruction/analysis program (OnDemand3D; CyberMed, Irvine, CA, USA), and the best image of the femur showing the lateral and medial epicondylar prominences was selected. In addition, a transverse image of the proximal tibia at the optimal osteotomy level (10 mm below the highest point of the lateral plateau) was also selected. The reference axis was defined as a line perpendicular to the surgical TEA (sTEA; a line from the tip of the lateral epicondyle to the sulcus of the medial epicondyle) of the femur (Fig. 1a). Tibial rotational alignment was measured as the angle between the reference axis and each of five anteroposterior (AP) axes, at the level of tibial osteotomy. The five axes were identified and defined as follows: (1) a line from the center of the posterior cruciate ligament (PCL) to the medial border of the patellar tendon (PCL-PT; Fig. 1b) [18]; (2) a line from the center of the PCL to the medial border of the tibial tuberosity (PCL-TT1; Fig. 1c) [19]; a line from the center of the PCL to the medial one-third of the tibial tuberosity (PCL-TT2; Fig. 1d) [20]; (4) a line from the center of the PCL to the apex of the tibial tuberosity (PCL-TT3; Fig. 1e) [27]; and (5) the AP axis of the tibial prosthesis, using a tibial prosthesis template (LPS-Flex; Zimmer, Warsaw, IN, USA), along the ATCC (Fig. 1f) [17]. Internal rotation was shown as a negative value, while external rotation was shown as a positive value.

### Statistical Analysis

A priori sample size analysis using G*Power version 3.1.2 showed that 30 cases per group were required to detect a statistically significant between-group difference with 1° precision in terms of tibial component rotation (α = 0.05, β = 0.8). For descriptive analysis, data are presented as mean values with standard deviations (SDs) and ranges. All data were tested for normal distribution using the Kolmogorov-Smirnov test. When a normal distribution was present, the Student’s *t* test was used to evaluate the relationship within each group, and the paired *t* test was used to examine the significance of the difference for each axis. Radiographic parameters were measured twice by two independent observers (JJ and JIK), with a two-week interval between measurements. Intra- and interobserver reliability were assessed using intraclass correlation coefficients (ICCs). Statistical analyses were performed using SPSS version 20.0 (IBM Corporation, Armonk, NY, USA), with *P* values of <0.05 considered significant.

## Results

For all five axes tested, the mean angles were smaller in OA knees than in normal knees (*P* < 0.05). The AP axes of the proximal tibia showed greater internal rotation in OA knees than in normal knees. The difference in the mean angle of the ATCC axis between OA and normal knees was smaller than that of the other axes tested (*P* = 0.047). In normal knees, the angle of the ATCC axis had the smallest mean value and narrowest range (1.6° ± 2.8°; range, −1.7°–7.7°). In OA knees, the angle of the PCL-TT1 axis (0.3° ± 5.5°; range, −19.7°–10.6°) had the smallest mean value, but the SD and range exceeded those of the angle of the ATCC axis (0.8° ± 2.7°; range, −7.9°–9.2°). The mean angle of the ATCC axis was larger than that of the PCL-TT1 axis, but the difference was not significant (*P* = 0.461). The angle of the ATCC axis had the smallest SD and narrowest range (Table 2). The ICCs for inter- and intraobserver reliability were >0.8 for all measurements, ranging from 0.81 to 0.92, which indicated that all measurements had good reliability.

**Table 2:** The tibial component rotational alignment in normal and OA knees

	Tibial Component Rotational Alignment				
	Normal knees (*n* = 40)		OA knees (*n* = 120)		
	Mean ± SD (°)^a^	Range (°)^a^	Mean ± SD (°)^a^	Range (°)^a^	*P* value^b^
PCL-PT	2.8 ± 4.9	−8.2 ~ 13.2	−1.2 ± 4.7	−13.8 ~ 10.0	< 0.001
PCL-TT1	4.0 ± 4.7	−6.2 ~ 12.2	0.3 ± 5.5	−19.7 ~ 10.6	< 0.001
PCL-TT2	14.7 ± 5.0	5.4 ~ 23.5	9.8 ± 5.4	−8.1 ~ 21.9	< 0.001
PCL-TT3	19.9 ± 5.4	8.4 ~ 28.7	14.8 ± 5.4	0 .3 ~ 27.8	< 0.001
ATCC	1.6 ± 2.8	−1.7 ~ 7.7	0.8 ± 2.7	−7.9 ~ 9.2	0.047

^a^Internal rotation was denoted as a negative value, and external rotation as a positive value

^b^Student’s *t-*test

## Discussion

The most important findings of this study were as follows: (1) the AP axes of the proximal tibia showed greater internal rotation in OA knees than in normal knees, and (2) although the mean angle of the ATCC axis was larger than that of the PCL-TT1 axis, the angle of the ATCC axis had the smallest SD and narrowest range noted for OA knees.

Based on previous reports, the nature of OA-related rotational deformity of the tibia with respect to the corresponding position in normal knees is debatable. In a CT study, Matsui et al. [30] reported that the tibia tends to be externally rotated in OA knees with varus deformity. Similarly, using magnetic resonance imaging, Sahin et al. [31] showed that, in OA knees with varus deformity, the tibia tends to rotate externally relative to the orientation noted in normal knees. On the contrary, in the present study, although the absolute values of the angles of the AP axes indicated external rotation compared with the reference axis (except for the PCL-PT axis), OA knees were found to show a tendency to rotate internally relative to the orientation noted in normal knees. This finding is consistent with that of Khan et al. [32], who showed greater external rotation in normal knees than in OA knees. This aspect is important clinically because, if information regarding tibial rotational alignment in normal knees is applied to OA knees, the tibial component might rotate externally and result in malrotation-related adverse outcomes after TKA.

Rotational alignment of the tibia is important for the long-term success and good functional outcome of TKA. However, the tibial component may malrotate intraoperatively due to a lack of distinct landmarks after cutting the tibia or anatomic variability [20, 21, 28]. Several authors reported that excessive internal rotation of the tibial component causes patellar maltracking and persistent anterior knee pain [5–7]. Other reports also mentioned flexion and mid-flexion instability due to poor matching between the tibial and femoral components through the range of motion [8–10]. Some authors expressed particular concern regarding the association between the malrotation of the tibial component and premature wear of the polyethylene components, leading to component loosening [11–13]. Moreover, Su et al. [33] reported that malrotation of the tibial component is one of the causes of knee stiffness, whereas Barrack et al. [2] reported that even small deviations (6.2°) towards internal rotation of the tibial component were associated with increased postoperative pain. Finally, the mechanism underlying the detrimental effect of malrotation of the tibial component was confirmed not only in clinical studies but also in a biomechanical study, which showed that malrotation is implicated in increased biomechanical stress induced by AP translations [34].

To determine the rotational alignment of the tibial component in TKA, several anatomical landmarks on the proximal tibia have been proposed [17–27]. Many studies have shown that the tibial tuberosity is a reliable landmark [24, 35–37]. However, there is some concern that employing the tibial tuberosity as a landmark results in malrotation of the tibial component. As for the medial one-third of the tibial tuberosity, Dalury et al. [19] reported that the tibial tray should be rotated externally to the medial one-third of the tibial tuberosity to maximize function. In addition, Eckhoff et al. [20] demonstrated that an average of 19° of external rotation of the tibial component relative to the femoral component occurred when the medial one-third of the tibial tuberosity was used as a reference for tibial rotational alignment. As for the medial border of the tibial tuberosity, Huddleston et al. [21] evaluated a neutral point on the rotating tibial insert and reported that this point was approximately 5° external to the medial border of the tibial tuberosity. In this study, the mean angles of these landmarks varied greatly compared with the others, indicating that isolated use of this landmark could result in tibial malrotation.

The PCL-PT axis also has been suggested as a reliable reference line for tibial rotational alignment [19]. This landmark was initially proposed by Akagi et al. [18], who reported that the mean angle between this line and a line perpendicular to the clinical epicondylar axis of the femur in normal knees was 0°, ranging from 6.3° of internal rotation to 5.2° of external rotation. Sahin et al. [31] also reported that Akagi’s line was the least affected by interobserver inconsistency, and, therefore, provided the best guidance for determining tibial rotational alignment. Our result using the PCL-PT axis as a landmark in OA knees (−1.2° ± 4.7°) is similar to reported findings [18, 24, 31, 38]; however, the SD was greater, indicating more variability than the angle of the ATCC axis (0.8° ± 2.7°).

In our study, we found that the ATCC axis had a narrow SD with the least variability. The mean angle of the ATCC axis (0.8° ± 2.7°) was larger than that of the PCL-TT1 axis (0.3° ± 5.5°), but the difference was not significant. Additionally, use of the ATCC as a landmark has several advantages over the other axes. First, a single area is more readily identifiable than a single point or line. The ATCC can be palpated after tibial cutting during TKA, and, thus, proper positioning of the tibial component can be achieved intraoperatively. Unfortunately, many sagittal axes are not easily identifiable during surgery. Second, if preoperative CT images are obtained to improve positioning of the tibial component, the ATCC can offer a simple and accurate method to predict tibial rotational alignment after TKA by applying the tibial prosthesis template on the CT image at the desired osteotomy level. Therefore, we believe that the ATCC is a reliable and useful anatomical landmark for tibial rotational alignment.

There are several limitations to our study. First, this study only addressed tibiofemoral conformity with the knee in extension, and not through the arc of flexion, during which the degree of matching may change. However, in their biplanar image-matching study, Asano et al. [14] reported that the flexion-extension axis of the knee corresponded to an sTEA of 0° to 90°. Because the present study compared the usefulness of five axes with reference to the sTEA, we believe that tibiofemoral conformity in flexion would be better with use of the ATCC axis because this axis has less deviation from the sTEA. Second, with the advent of mobile bearings systems and customized devices, the importance of landmarks for rotation alignment of tibia may decreased. However, even with mobile bearing systems, obtaining accurate tibial component rotation remains of key importance because substantial malrotations may not be entirely corrected using mobile bearing systems. Thus, it is more feasible to obtain tibial component rotation as precise as possible, and correct some minor errors by mobile bearing systems. Customization has certain disadvantages such as increased inconvenience and economic cost. Therefore, rather than implementing customization in routine clinical practice, we believe that it is better to use customized devices only when severe deformity exists. The ATCC-based method for determining tibial rotational alignment is simple and accurate, because the ATCC can be palpated after tibial cutting and, thus, proper positioning of the tibial component can be achieved easily even without customized devices.

Finally, the number of normal knees was small compared with the number of OA knees, and the patients’ characteristics such as age and mechanical tibiofemoral angle were not matched between the groups. However, OA is more prevalent at older ages, while normal knees are rarer; therefore, it is difficult to match the age. In addition, although the mechanical tibiofemoral angles were not matched between groups, it should be noted that, in each group, the angles of five axes were compared under the same mechanical tibiofemoral angle. Therefore, we believe that ATCC is the most accurate landmark for determining the rotational alignment of the tibia in both normal and mild-to-moderate varus alignment.

## Conclusion

In this study, we compared the usefulness of five anatomical landmarks on the proximal tibia for determining tibial rotational alignment in TKA. Compared to the observations in normal knees, these AP axes showed greater internal rotation relative to sTEA in OA knees. The ATCC was found to be the most reliable and useful anatomical landmark for determining tibial rotational alignment in TKA.

## Abbreviations

AP: Anteroposterior
ATCC: Anterior tibial curved cortex
CT: Computed tomography
OA: Osteoarthritic
PCL: Posterior cruciate ligament
PT: Patellar tendon
SD: Standard deviation
sTEA: Surgical transepicondylar axis
TKA: Total knee arthroplasty
TT: Tibial tuberosity

## References

[1] Insall JN, Kelly M (1986). The total condylar prosthesis. Clin Orthop Relat Res.

[2] Barrack RL, Schrader T, Bertot AJ, Wolfe MW, Myers L (2001). Component rotation and anterior knee pain after total knee arthroplasty. Clin Orthop Relat Res.

[3] Mulhall KJ, Ghomrawi HM, Scully S, Callaghan JJ, Saleh KJ (2006). Current etiologies and modes of failure in total knee arthroplasty revision. Clin Orthop Relat Res.

[4] Ritter MA, Lutgring JD, Davis KE, Berend ME (2008). The effect of postoperative range of motion on functional activities after posterior cruciate-retaining total knee arthroplasty. J Bone Joint Surg Am.

[5] Akagi M, Matsusue Y, Mata T, Asada Y, Horiguchi M, Iida H, et al. Effect of rotational alignment on patellar tracking in total knee arthroplasty. Clin Orthop Relat Res. 1999;366:155–63. 10.1097/00003086-199909000-00019 10627729

[6] Nagamine R, Whiteside LA, White SE, McCarthy DS (1994). Patellar tracking after total knee arthroplasty. The effect of tibial tray malrotation and articular surface configuration. Clin Orthop Relat Res.

[7] van Gennip S, Schimmel JJ, van Hellemondt GG, Defoort KC, Wymenga AB (2014). Medial patellofemoral ligament reconstruction for patellar maltracking following total knee arthroplasty is effective. Knee Surg Sports Traumatol Arthrosc.

[8] Anouchi YS, Whiteside LA, Kaiser AD, Milliano MT (1993). The effects of axial rotational alignment of the femoral component on knee stability and patellar tracking in total knee arthroplasty demonstrated on autopsy specimens. Clin Orthop Relat Res.

[9] Romero J, Duronio JF, Sohrabi A, Alexander N, MacWilliams BA, Jones LC (2002). Varus and valgus flexion laxity of total knee alignment methods in loaded cadaveric knees. Clin Orthop Relat Res..

[10] Van Damme G, Defoort K, Ducoulombier Y, Van Glabbeek F, Bellemans J, Victor J (2005). What should the surgeon aim for when performing computer-assisted total knee arthroplasty?. J Bone Joint Surg Am.

[11] Hofmann S, Romero J, Roth-Schiffl E, Albrecht T (2003). [Rotational malalignment of the components may cause chronic pain or early failure in total knee arthroplasty]. Orthopade.

[12] Lewis P, Rorabeck CH, Bourne RB, Devane P (1994). Posteromedial tibial polyethylene failure in total knee replacements. Clin Orthop Relat Res.

[13] Wasielewski RC, Galante JO, Leighty RM, Natarajan RN, Rosenberg AG (1994). Wear patterns on retrieved polyethylene tibial inserts and their relationship to technical considerations during total knee arthroplasty. Clin Orthop Relat Res.

[14] Asano T, Akagi M, Nakamura T (2005). The functional flexion-extension axis of the knee corresponds to the surgical epicondylar axis: in vivo analysis using a biplanar image-matching technique. J Arthroplast.

[15] Churchill DL, Incavo SJ, Johnson CC, Beynnon BD (1998). The transepicondylar axis approximates the optimal flexion axis of the knee. Clin Orthop Relat Res.

[16] Miller MC, Berger RA, Petrella AJ, Karmas A, Rubash HE (2001). Optimizing femoral component rotation in total knee arthroplasty. Clin Orthop Relat Res.

[17] Baldini A, Indelli PF, DEL L, Mariani PC, Marcucci M (2013). Rotational alignment of the tibial component in total knee arthroplasty: the anterior tibial cortex is a reliable landmark. Joints.

[18] Akagi M, Oh M, Nonaka T, Tsujimoto H, Asano T, Hamanishi C (2004). An anteroposterior axis of the tibia for total knee arthroplasty. Clin Orthop Relat Res.

[19] Dalury DF (2001). Observations of the proximal tibia in total knee arthroplasty. Clin Orthop Relat Res.

[20] Eckhoff DG, Metzger RG, Vandewalle MV (1995). Malrotation associated with implant alignment technique in total knee arthroplasty. Clin Orthop Relat Res.

[21] Huddleston JI, Scott RD, Wimberley DW (2005). Determination of neutral tibial rotational alignment in rotating platform TKA. Clin Orthop Relat Res.

[22] Siston RA, Goodman SB, Patel JJ, Delp SL, Giori NJ (2006). The high variability of tibial rotational alignment in total knee arthroplasty. Clin Orthop Relat Res.

[23] Ikeuchi M, Yamanaka N, Okanoue Y, Ueta E, Tani T (2007). Determining the rotational alignment of the tibial component at total knee replacement: a comparison of two techniques. J Bone Joint Surg Br.

[24] Incavo SJ, Coughlin KM, Pappas C, Beynnon BD (2003). Anatomic rotational relationships of the proximal tibia, distal femur, and patella: implications for rotational alignment in total knee arthroplasty. J Arthroplast.

[25] Sahin N, Atici T, Kurtoglu U, Turgut A, Ozkaya G, Ozkan Y (2012). Centre of the posterior cruciate ligament and the sulcus between tubercle spines are reliable landmarks for tibial component placement.

[26] Sun T, Lu H, Hong N, Wu J, Feng C (2009). Bony landmarks and rotational alignment in total knee arthroplasty for Chinese osteoarthritic knees with varus or valgus deformities. J Arthroplast.

[27] Berger RA, Crossett LS, Jacobs JJ, Rubash HE (1998). Malrotation causing patellofemoral complications after total knee arthroplasty. Clin Orthop Relat Res.

[28] Akagi M, Mori S, Nishimura S, Nishimura A, Asano T, Hamanishi C (2005). Variability of Extraarticular Tibial Rotation References for Total Knee Arthroplasty. Clinical Orthopaedics and Related Research.

[29] Kim D, Seong SC, Lee MC, Lee S (2012). Comparison of the tibiofemoral rotational alignment after mobile and fixed bearing total knee arthroplasty. Knee Surg Sports Traumatol Arthrosc.

[30] Matsui Y, Kadoya Y, Uehara K, Kobayashi A, Takaoka K (2005). Rotational deformity in varus osteoarthritis of the knee: analysis with computed tomography. Clin Orthop Relat Res.

[31] Sahin N, Atici T, Ozturk A, Ozkaya G, Ozkan Y, Avcu B (2012). Accuracy of anatomical references used for rotational alignment of tibial component in total knee arthroplasty. Knee Surg Sports Traumatol Arthrosc.

[32] Khan MS, Seon JK, Song EK (2012). Rotational profile of lower limb and axis for tibial component alignment in varus osteoarthritic knees. J Arthroplast.

[33] Su EP, Su SL, Della Valle AG (2010). Stiffness after TKR: how to avoid repeat surgery. Orthopedics.

[34] Thompson JA, Hast MW, Granger JF, Piazza SJ, Siston RA (2011). Biomechanical effects of total knee arthroplasty component malrotation: a computational simulation. J Orthop Res.

[35] Coughlin KM, Incavo SJ, Churchill DL, Beynnon BD (2003). Tibial axis and patellar position relative to the femoral epicondylar axis during squatting. J Arthroplast.

[36] Graw BP, Harris AH, Tripuraneni KR, Giori NJ (2010). Rotational references for total knee arthroplasty tibial components change with level of resection. Clin Orthop Relat Res.

[37] Uehara K, Kadoya Y, Kobayashi A, Ohashi H, Yamano Y (2002). Bone anatomy and rotational alignment in total knee arthroplasty. Clin Orthop Relat Res.

[38] Aglietti P, Sensi L, Cuomo P, Ciardullo A (2008). Rotational position of femoral and tibial components in TKA using the femoral transepicondylar axis. Clin Orthop Relat Res.

